# The emerging role and mechanism of HMGA2 in breast cancer

**DOI:** 10.1007/s00432-024-05785-4

**Published:** 2024-05-16

**Authors:** Qing Ma, Sisi Ye, Hong Liu, Yu Zhao, Wei Zhang

**Affiliations:** 1 General Practice Ward/International Medical Center Ward, General Practice Medical Center, West China Hospital, Sichuan University /West China School of Nursing, Sichuan University, Chengdu, China; 2 Emergency Department of West China Hospital, Sichuan University/West China School of Nursing, Sichuan University, Chengdu, China

**Keywords:** Breast cancer, HMGA2, Metastasis, Non-coding RNAs, Drug resistance

## Abstract

High mobility group AT-hook 2 (HMGA2) is a member of the non-histone chromosomal high mobility group (HMG) protein family, which participate in embryonic development and other biological processes. HMGA2 overexpression is associated with breast cancer (BC) cell growth, proliferation, metastasis, and drug resistance. Furthermore, HMGA2 expression is positively associated with poor prognosis of patients with BC, and inhibiting HMGA2 signaling can stimulate BC cell progression and metastasis. In this review, we focus on HMGA2 expression changes in BC tissues and multiple BC cell lines. Wnt/β-catenin, STAT3, CNN6, and TRAIL-R2 proteins are upstream mediators of HMGA2 that can induce BC invasion and metastasis. Moreover, microRNAs (miRNAs) can suppress BC cell growth, invasion, and metastasis by inhibiting HMGA2 expression. Furthermore, long noncoding RNAs (LncRNAs) and circular RNAs (CircRNAs) mainly regulate HMGA2 mRNA and protein expression levels by sponging miRNAs, thereby promoting BC development. Additionally, certain small molecule inhibitors can suppress BC drug resistance by reducing HMGA2 expression. Finally, we summarize findings demonstrating that HMGA2 siRNA and HMGA2 siRNA-loaded nanoliposomes can suppress BC progression and metastasis.

## Introduction

Breast cancer (BC) is the most frequent invasive cancer in women, affecting millions of worldwide, and a leading cause of cancer-related death in women, second only to lung cancer (Barzaman et al. 2020; Jokar et al. 2021; Lacey et al. 2002). The incidence of BC in 2021 was approximately 85 per 100,000 women (Han et al. 2013; Ferlay et al. 2015). Based on incidence and mortality rates, the global burden of BC is increasing profoundly (Coughlin 2019; Coughlin and Ekwueme 2009). Numerous endogenous and exogenous factors are associated with BC risk, including increased age, gene mutation, family history, early menarche, delayed menopause, and mammography density (Bodewes et al. 2022; Starek-Świechowicz et al. 2021). The prognosis of patients with early-stage BC is relatively good, while that of individuals with metastatic disease is poor (Harbeck et al. 2019; Valastyan and Weinberg 2011). BC can be divided into three groups, according to its molecular characteristics: estrogen receptor (ER) or progesterone receptor (PR) positive, human epidermal receptor 2 (HER2) positive, and triple-negative BC (TNBC; ER − , PR − , HER2 −), of which TNBC is the most common (Barzaman et al. 2020; Liedtke et al. 2023; Lehmann et al. 2011; Li et al. 2021). Although BC diagnosis and treatment have improved significantly, there remains an urgent need to identify new biomarkers and therapeutic targets, and to elucidate the potential mechanisms involved in BC infiltration and metastasis (Ye et al. 2023). Further, while the five-year survival rate of patients with BC has gradually improved, tumor cell drug resistance, caused by radiotherapy and chemotherapy, eventually leads to the development of more aggressive tumors (Zhao et al. 2021a,b; Zhang et al. 2021; Ellis and Hicklin 2009). Most BC-related deaths are caused by metastasis to other organs, rather than the primary tumor itself (Zhang et al. 2021; Weigelt et al. 2005).

Metastasis is a complex and multi-step process, with steps including invasion, infiltration, and colonization of distant organs (Clark and Vignjevic 2015). Further, cells must undergo phenotypic changes to adapt to the constantly changing microenvironment and external alterations during metastasis (Nieto et al. 2016). The most prominent type of cell phenotype change is epithelial-mesenchymal transition (EMT), which can promote cancer cell invasion, infiltration, and colonization of distant organs (Cui et al. 2023). Furthermore, the tumor microenvironment (TME) creates an ecological niche for interactions among tumor cells, surrounding endothelial cells, and fibroblasts, which can induce tumor cell proliferation, angiogenesis, and metastasis, and has an indispensable role in BC development (Deepak et al. 2020; Tahmasebi Birgani and Carloni 2017). Chronic inflammation is also an important cause of cancer development (Maurya et al. 2024; Jin et al. 2022). Pro-inflammatory factors secreted by the TME can promote epigenetic changes in chromatin, further inducing tumor formation and metastasis (Maimon et al. 2021; Elsässer et al. 2011). Hence, the mechanisms underlying BC development are complex, and it is urgent to explore the molecular processes involved in promotion of BC progression, to identify new biomarkers for accurate prediction of patient prognosis and development of molecular targeted therapies.

## Biological role of HMGA2

Transcription factors are proteins with domains that bind to the promoter or enhancer regions of DNA in their target genes (Ma et al. 2020). High mobility group AT-hook 2 (HMGA2) is a member of the non-histone chromosomal high mobility group (HMG) protein family, and the gene encoding HMGA2 maps to human chromosome 12q14–15 and mouse chromosome 10 (Kang et al. 2014; Huang et al. 2021). HMGA2 expression levels are minimal in normal tissues, but significantly elevated in disease models, and particularly in tumor tissues (Kaur et al. 2016; Niu et al. 2019; Huang et al. 2022a, b). HMGA2 selectively binds to AT-rich DNA sequences through its unique structural feature, AT hook DNA-binding motifs, thereby regulating DNA transcription (Zhao et al. 2020). Under stimulation with chemotherapy drugs, HMGA2 exhibits deoxyribosyl phosphate/apurinic/apyrimidinic (dRP/AP) site cleavage activity, thereby promoting cell resistance to DNA damage targeted by the base excision repair pathway (Summer et al. 2009). The fork chaperone function of HMGA2 suppresses competitive inhibitory peptide-induced DNA damage, thereby preserving genome integrity in stem and cancer cells (Yu et al. 2014). HMGA2 is involved in numerous biological processes, including embryonic development, inflammatory responses, apoptosis, and cell aging (Ashar et al. 2010). Further, HMGA2 can inhibit osteogenic differentiation of mesenchymal stem cells and impede new bone regeneration. In addition, HMGA2 is reported to participate in Alzheimer’s disease pathogenesis, by affecting intracranial volume (Huang et al. 2021; Stein et al. 2012; Tang et al. 2019).

Most studies have confirmed that HMGA2 is established to be a multifunctional regulatory factor that promotes tumor occurrence and differentiation, and maintains cell stemness (Fusco and Fedele 2007; Song et al. 2021), In the context of malignancies, HMGA2 is highly expressed in numerous tumors, including colon cancer, BC, lung cancer, ovarian cancer, prostate cancer, and oral squamous cell carcinoma (Han et al. 2023; Hawsawi et al. 2018; Wang et al. 2021a, b, c). Upregulated HMGA2 expression can be attributed to rearrangement of chromosome 12q14–15 or DNA hypomethylation at the HMGA2 genome locus (George et al. 2019). Hence, HMGA2 has important roles in various diseases, including tumors, and its regulatory function warrants further exploration. There is increasing evidence suggesting that transcriptional regulation mediated by HMGA2 is a key factor in BC metastasis (Sun et al. 2013), and deeper understanding of the molecular mechanisms underlying BC has led to the recognition of specific targets (Velikyan 2020), which can be used to diagnose and treat individuals, thereby improving treatment efficacy (Jokar et al. 2021). The aims of this review were to explore the mechanisms involved in HMGA2 activity in BC development and metastasis, as well as summarizing the latest research advances.

## HMGA2 expression levels in BC

Recently, involvement of HMGA2 in BC has been extensively reported. Several studies have reported that *HMGA2* mRNA levels are clearly higher in tumors than those in adjacent tissues from patients with BC (Zhao et al. 2018; Mansoori et al. 2021; Wu et al. 2021). Wu et al. reported that HMGA2 expression levels can serve as a prognostic marker, since they are related to the stage of BC (Wu et al. 2016). Furthermore, higher HMGA2 levels are significantly correlated with advanced tumor grade, lower survival rate, and poor prognosis in patients with BC (Mansoori et al. 2021). In addition, many BC cell types show elevated HMGA2 expression levels; HMGA2 expression is reported to be significantly upregulated in BC cell lines (MDA-MB-231, SUM149, and BT549) relative to MCF10A normal breast epithelial cells, which are often used as negative control cells (Xu et al. 2021).

Furthermore, HMGA2 protein levels in MDA-MB-231 and MDA-MB-453 cells are significantly higher than those in MCF-10A cells, and further up-regulated in drug-resistant BC cells (Zhu et al. 2021). Moreover, HMGA2 expression is higher in MCF-7 and MDA-MB-231 cells than that in MCF10A cells (Wu et al. 2021; Yang et al. 2022). Other than MCF-7 and MDA-MB-231 cells, there are also reports that HMGA2 expression levels are clearly elevated in BT-549 and MDA-MB-453 cells relative to those in MCF10A cells (Wang et al. 2019). Overall, HMGA2 overexpression in patients with BC and BC cells appears to be closely related to tumor progression (Table 1).

**Table 1:** HMGA2 expression levels in breast cancer

Tumor samples and cells	Normal samples and cells	HMGA2 expression level (BC/NC)	References
Tumor tissue from patients with BC (n = 24)	Adjacent normal tissue (n = 24)	Upregulated mRNA levels	Mansoori et al. (2021)
MDA-MB-231, SUM149, and BT549 cells	MCF-10A, T47D, SKBR3, and BT474 cells	Upregulated protein levels	Xu et al. (2021)
MDA-MB-231 and MDA-MB-453 cells	MCF-10A cells	Upregulated protein levels	Zhu et al. (2021)
Tumor tissue from patients with BC (n = 64); MCF7 and MDA-MB-231 cells	Adjacent normal tissue (n = 64); MCF10A cells	Upregulated protein and mRNA levels	Yang et al. (2022)
Tumor tissue from patients with BC (n = 68); MCF-7, MDA-MB-231, and MDA-MB-468 cells	Adjacent normal tissue (n = 68); MCF10A cells	Upregulated protein and mRNA levels	Yin et al. (2021)
Tumor tissue from patients with BC (n = 30); MCF7 and MDA-MB-231 cells	Adjacent normal tissue; MCF10A cells	Upregulated protein and mRNA levels	Wu et al. (2021)
MCF-7, BT-549, MDA-MB-231, and MDA-MB-453 cells	MCF10A cells	Upregulated protein levels	Wang et al (2019)
Tumor tissue from patients with BC (n = 20)	Adjacent normal tissue (n = 20)	Upregulated mRNA levels	Zou et al. (2016)
Tumor tissue from patients with BC (n = 24)	Adjacent normal tissue (n = 24)	Upregulated mRNA levels	Mansoori et al. (2019)

*BC* Breast cancer, *NC* Negative control

### Functions of HMGA2 in BC

BC is a highly heterogeneous disease, and tumor invasion and distant metastases are the events that lead to the majority of BC-related deaths (Wu et al. 2019; Saha et al. 2021). Most BC patients with distant metastasis exhibits clear organ preference, most frequently occurring in brain, lung, liver, lymphatic, and bone tissues (Wang et al. 2021a, b, c; Ehrenfeld et al. 2019). There is increased evidence that HMGA2 functions as a critical factor in the progression and anti-drug resistance of various tumors by influencing processes including tumor growth, infiltration, metastasis, and apoptosis (Campos Gudiño et al. 2023; Chen et al. 2019; Yu et al. 2021). Immunohistochemistry staining of HMGA2 in 30 TNBC tumor samples demonstrated that it can contribute to lymph node metastasis (Tang et al. 2018). In a cell model, HMGA2 overexpression stimulates invasion and metastasis and is accompanied by up-regulation of VEGFR, Snail-1, and Smad3 (Mansoori et al. 2020). Some signaling proteins and non-coding RNAs contribute to regulation of HMGA2-induced BC infiltration and metastasis. For example, CCN6, a matrix-associated protein secreted by breast epithelial cells, mediates crosstalk between epithelial cells and the extracellular matrix, playing a critical role in the development and maintenance of EMT progression (Kleer et al. 2007; Pal et al. 2012).

## Signaling proteins

**Fig. 1:**
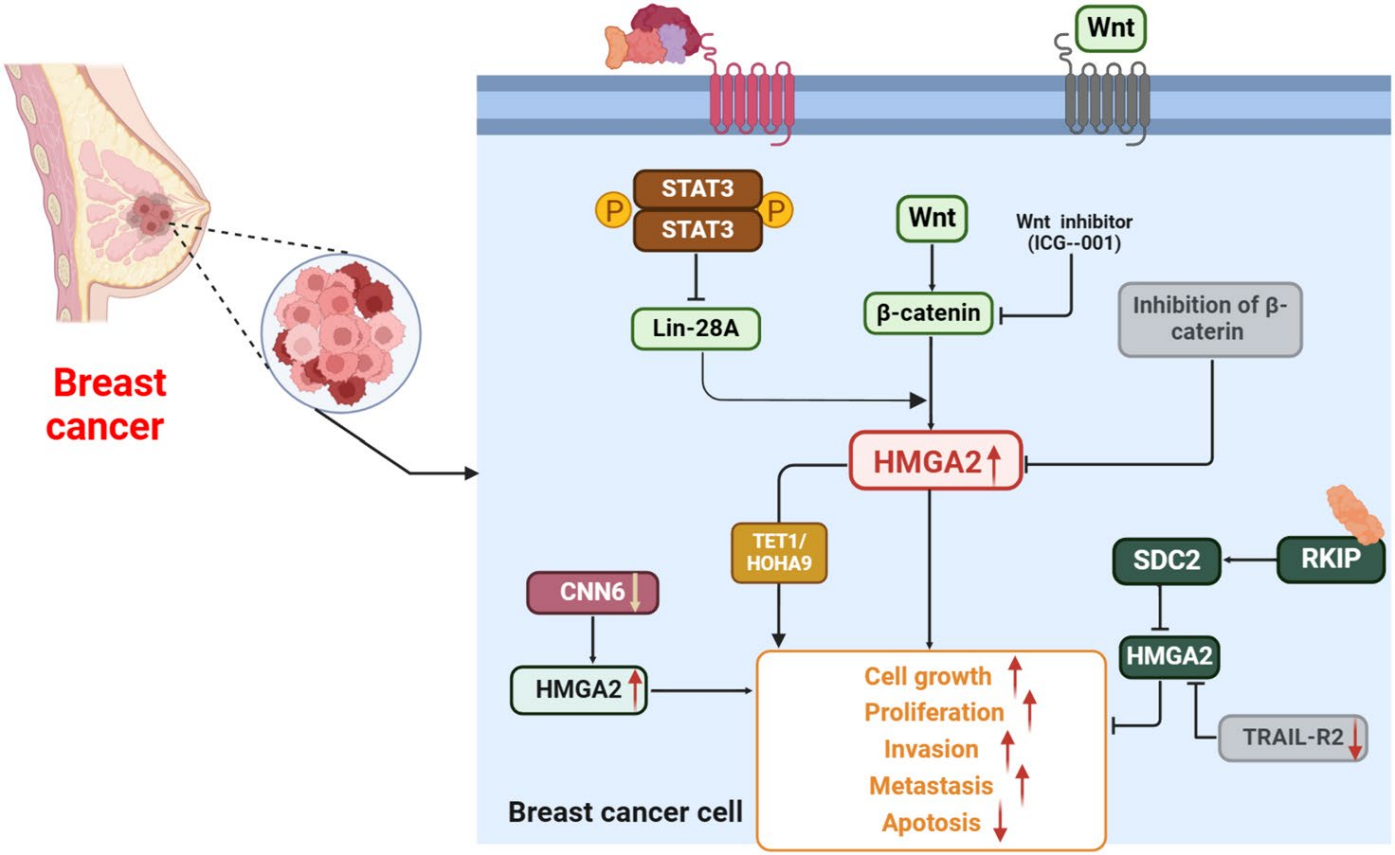
HMGA2 is involved in BC malignant progression through various signaling pathways. The Wnt/β-catenin, STAT3, CNN6, and TRAIL-R2 proteins are upstream mediators of HMGA2 that can induce BC cell growth, proliferation, invasion, and metastasis. The Wnt inhibitor, ICG-001, impedes β-catenin transcription and *HMGA2* mRNA expression in TNBC cells. Similarly, inhibiting β-catenin signaling in TBNC cells suppresses HMGA2 expression; however, RKIP depletes syndecan-2 (SDC2) levels, leading to suppression of invasion by decreasing HMGA2 expression. In addition, some key downstream mediators of HMGA2 have been reported to regulate BC invasion and metastasis, including the HMGA2-TET1-HOXA9 signaling pathway

Wnt signaling is a relatively evolutionarily conserved pathway in animals (Xu et al. 2020a, b). A growing number of studies have clarified that Wnt signaling participated in invasion, metastasis, and drug resistance (Wend et al. 2013; Malladi et al. 2016; Merikhian et al. 2021). Further, Wnt signaling activation is a critical factor in BC invasion and metastasis (Wellenstein et al. 2019). Recent research has provided evidence supporting a role for the Wnt-10B axis (β-catenin/HMGA2/EZH2) in survival and metastasis of patients with TNBC, and found that the Wnt inhibitor, ICG-001, impedes β-catenin transcription and *HMGA2* mRNA expression in TNBC cells, suggesting that HMGA2 is a downstream target in Wnt-10B-related metastasis (Fatima et al. 2019). Similarly, another study found that both Wnt10B and HMGA2 are highly expressed in a subset of TNBC tumors, and correlated with poor patient survival outcomes (Wend et al. 2013). Inhibition of β-catenin signaling in TBNC cells disrupts the interaction among LEF-1, TCF4, and CBP, suppressing HMGA2 expression (Wend et al. 2013). Further, reduction of CCN6 expression, together with higher IGF2BP2 and/or HMGA2 levels, can be a marker and regulator of metaplastic carcinomas of the breast (McMullen et al. 2018). Raf-1 kinase inhibitory protein (RKIP/PEBP1) suppresses metastasis and is associated with survival prognosis in patients with many types of tumors (Yun et al. 2011; Sun et al. 2023; Heuvelings et al. 2023). Sun et al. used both human BC cells and an MMTV-Wnt mouse BC model to demonstrate that RKIP depletes syndecan-2, leading to suppression of invasion by decreasing HMGA2 expression (Sun et al. 2014). TNF-related apoptosis inducing ligand receptor 2 (TRAIL-R2), is a TNF receptor family (Pan et al. 1997). The research showed that the knockdown of TRAIL-R2 diminished the HMGA2, p-Akt and CXCR4 expression, thereby suppressing diminish of the skeletal metastasis in BC (Pan et al. 1997; Azijli et al. 2013). In addition, key downstream mediators of HMGA2 have been identified as potential regulators of BC invasion and metastasis. Coordinate regulation of the HMGA2-TET1-HOXA9 signaling pathway may be a marker of survival and prognosis of patients with BC (Sun et al. 2013). Furthermore, combined knockdown of both HMGA2 and Bach-1 diminished BC cell proliferation, migration, and EMT progression (Mansoori et al. 2019). Importantly, Stat3 represses Lin-28 transcription and increases HMGA2 expression, effectively leading to EMT progression in BC (Guo et al. 2013) (Fig. 1).

## Non-coding RNAs

**Fig. 2:**
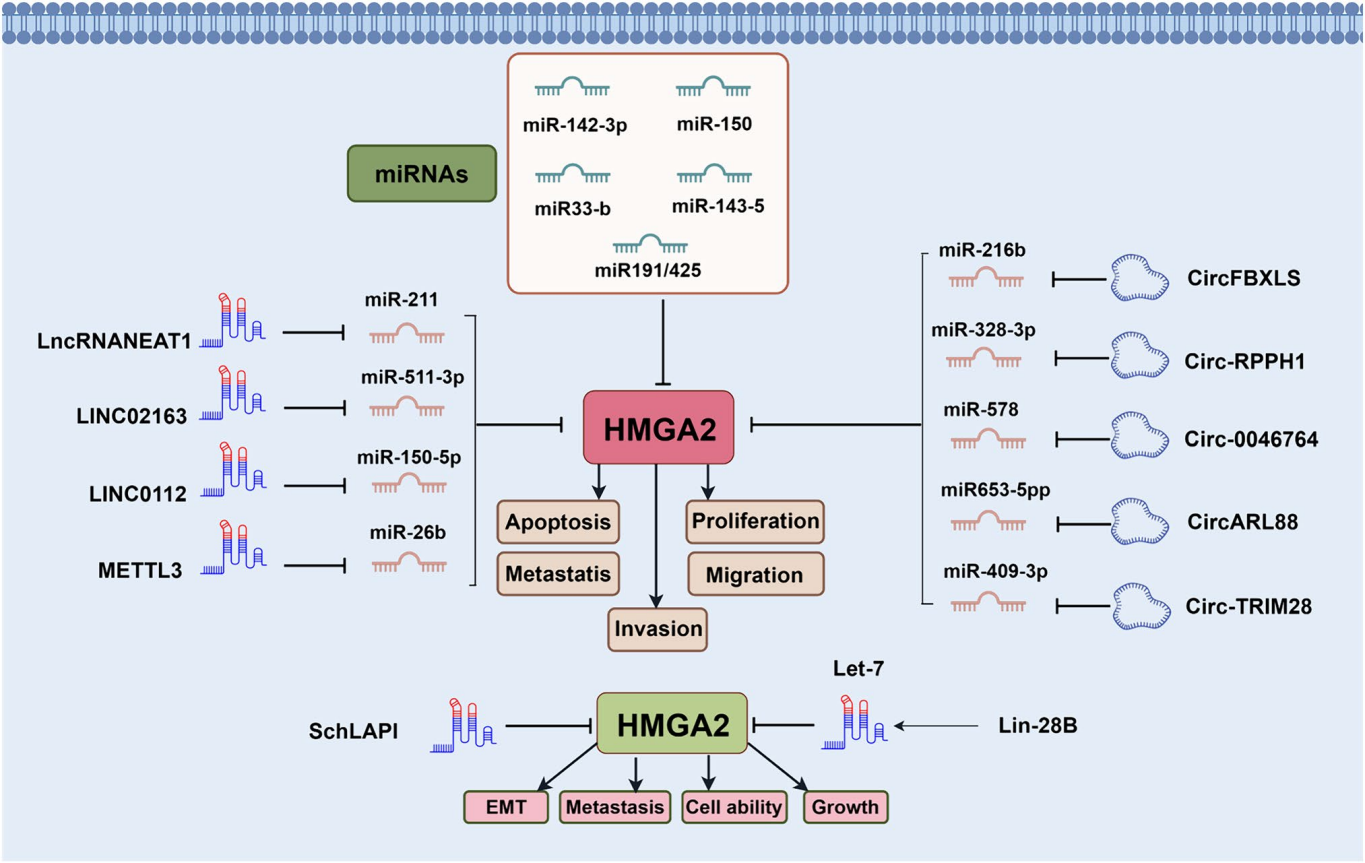
The non-coding RNA/HMGA2 axis in BC. miRNAs can suppress BC cell growth, invasion, and metastasis by inhibiting HMGA2 expression. Further, lncRNAs and circRNAs regulate HMGA2 mRNA and protein levels by sponging miRNAs, thereby promoting tumor development, apoptosis, proliferation, invasion, migration, and metastasis

Non-coding RNAs are critical molecules in BC progression, and interactions between non-coding RNAs and HMGA2 that influence BC progression have been well-studied (Fig. 2).

### MicroRNAs (miRNAs)

Tumor invasion, infiltration, and invasion and colonization to distant sites are regulated by gene expression changes, including via miRNAs. Similarly, miRNAs participate in BC development and metastasis by regulating the expression of target genes (Petri and Klinge 2020; Chen et al. 2012). The research demonstrated that miR-142-3p directly targeted the 3 ‘untranslated region of HMGA2, thereby down-regulating HMGA2 protein and mRNA expression levels, inhibiting BC cell dryness and reducing apoptosis, suggesting that HMGA2 is a direct target of miR-142-3p (Mansoori et al. 2021). In TNBC, miR-150 expression is downregulated, which reduces its suppression of lymph node metastasis through its effects in decreasing HMGA2 expression (Tang et al. 2018). As a negative regulatory molecule, miR-33b impeded the migration and invasion of BC and MCF-10A cells by influencing HMGA2 and Twist1 levels (Lin et al. 2015). Another study suggested that miR-143–5 suppresses BC progression though direct regulation of HMGA2 (Mansoori et al. 2023). Furthermore, the miR-191/425/DICER1/let-7/HMGA2 axis functions in the progression and invasion of BC cells and the MDA-MB-231 cell line (Zhang et al. 2018). Cancer stem cells (CSCs) are a minority subpopulation among malignant tumor cells, also referred to as tumor-initiating cells, which have important roles in metastatic dissemination, and treatment resistance (Clara et al. 2020). Guo et al. found that M1 macrophages contribute to CSC phenotypic transformation through the Lin-28B-let-7-HMGA2 axis, and HMGA2 expression inhibition directly reversed levels of proinflammatory signals, indicating that HMGA2 functions as a determinant factor in this pathway (Guo et al. 2019).

### Long non-coding RNAs (lncRNAs)

The biological function of lncRNAs in BC has been widely reported (Liu et al. 2021). Aberrant lncRNA expression significantly promotes TNBC cell proliferation, metastasis, and tumorigenicity (Niu et al. 2019; Xu et al. 2020a, b; Wang et al. 2018). Current evidence suggests that LINC02163 overexpression has a carcinogenic role, while the miR-511-3p/HMGA2 axis participates in the pro-oncogenic activities of LINC02163 in BC (MDA-MB-231 and MCF-7) cells (Qin et al. 2020). Wang et al. found that LINC01121 represents a molecular target for promoting BC cell proliferation, migration, and metastasis by regulating the miR-150-5p/HMGA2 signaling axis (Wang et al. 2019). In addition, SchLAP1 knockdown inhibits HMGA2 mRNA and protein expression in TNBC cells; however, HMGA2 overexpression could reverse the decrease in TNBC cells caused by HK2 knockdown (Bai et al. 2021). The RNA modification method, N6-methyladenine (m6A), occurs in almost all eukaryotes (Yin et al. 2021; Wei et al. 2017), and METTL3 is an m6A methyltransferase complex catalytic subunit that participates in numerous biological processes (Wang et al. 2014). Aberrant m6A modification occurs in various types of cancer, including BC (Zhang et al. 2016). Recent research demonstrated that m6A methyltransferase METTL3 was shown to up-regulate MALAT1 expression, and high MALATA3 expression promotes EMT progression, migration, and invasion in BC by targeting the MALAT1/miR-26b/HMGA2 axis (Zhao et al. 2021a, b). Further, overexpression of the lncRNANEAT1, contributes to malignant tumor proliferation (Li et al. 2017), and lncRNANEAT1 can contribute to cell metastasis by negatively regulating the miR-211/HMGA2 axis.

### Circular RNAs (circRNAs)

CircRNAs are a group of circular non coding RNAs that regulate mRNA levels by competing with miRNAs, thereby participating in the occurrence and development of various cancers (Yao et al. 2022; Huang et al. 2022a, b). CircRNAs regulate HMGA2 expression by competing miRNAs, thus promoting BC development (Zhu et al. 2021). For example, circFBXL5 can promote 5-FU resistance of BC by inducing MDA-MB-231 and MDA-MB-453 cell invasion and apoptosis, and the miR-216b/HMGA2 axis is involved in this process, suggesting that circFBXL5 can sponge miR-216b to promote HMGA2 expression (Zhu et al. 2021). Furthermore, Circ-RPPH1 expression levels are raised in BC tissues and cells, while circ-RPPH1 knockdown alleviates the EMT phenotype and invasion by targeting the miR-328-3p/HMGA2 axis, representing a potential BC signaling pathway (Li et al. 2022). In addition to Circ-RPPH1, Circ_0048764 also stimulates BC cell proliferation, migration, and invasion by regulating miR-578 and increasing HMGA2 expression (Ding et al. 2023). Furthermore, circARL8B overexpression eliminates the inhibitory effects of miR-653-5p on BC (MCF-7 and MDA-MB-231) cell development, while circARL8B knockdown restrained BC cell viability, invasion, and fatty acid metabolism by regulating miR-653-5p/HMGA2 signaling (Wu et al. 2021). In MCF7/R and MDA-MB-231 cells, HMGA2 overexpression enhances cell invasion ability, inhibits apoptosis, and restores the influence of sh-circTRIM28, suggesting that sh-circTRIM28 inhibition can restore BC progression by targeting miR-409-3p/HMGA2 signaling (Yang et al. 2022).

## Therapeutic and delivery strategies targeting HMGA2 in BC

**Fig. 3:**
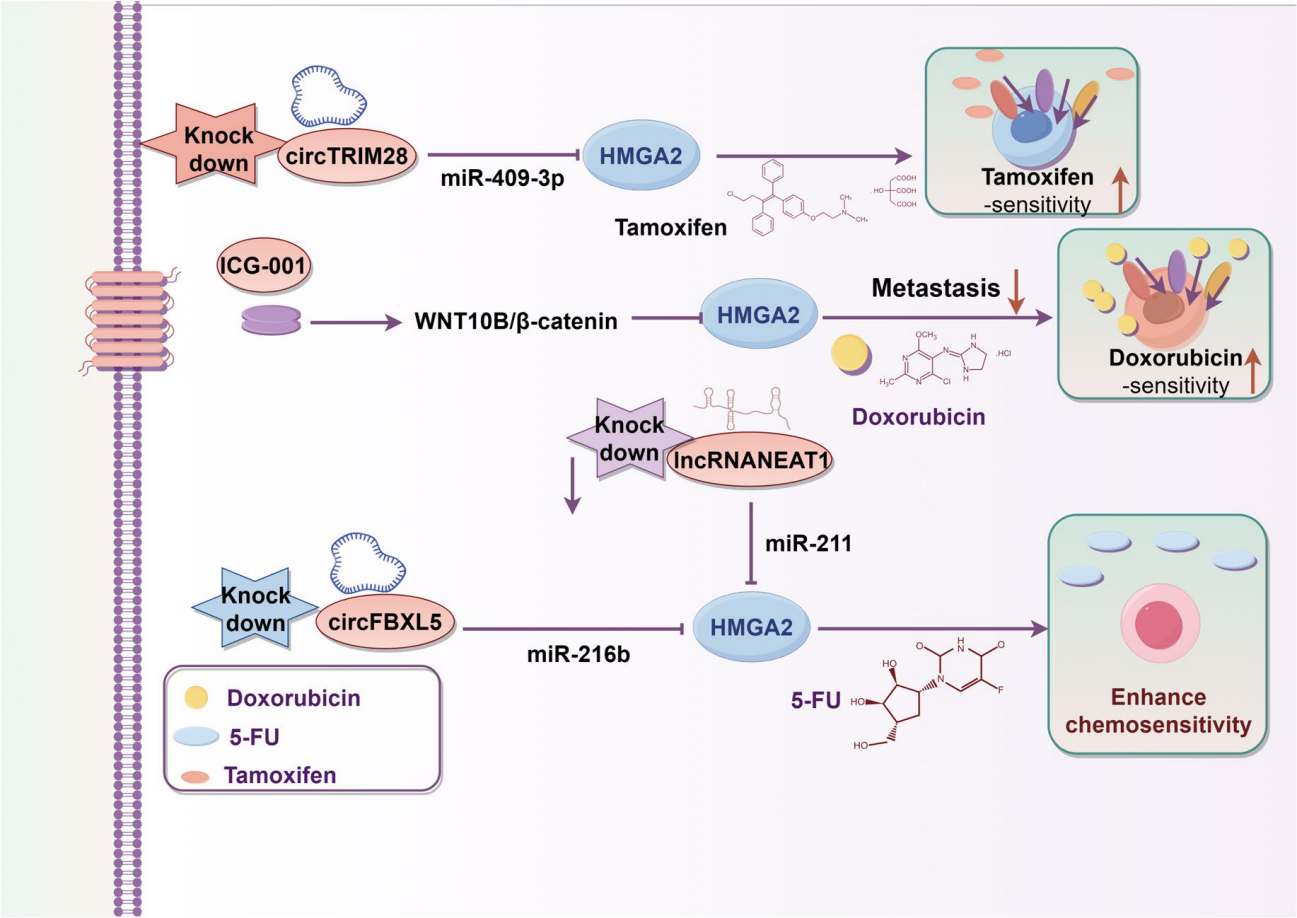
Summary of studied strategies of HMGA2-based for BC treatment. The figure shows the strategies that directly or indirectly downregulate HMGA2 expression for BC treatment, including small molecule inhibitors and miRNA/HMGA2 axis

HMGA2 is considered to be an important participant in BC progression. These processes are related to the induction of chemoresistance. Thus, strategies that directly or indirectly downregulate HMGA2 functions have become the focus of treatment in BC (Fig. 3).

## Small molecule inhibitors

In clinical practice, conventional drugs, such as chemotherapy, hormone, and targeted drugs, are used as first-line tumor treatment methods (Jayaraj et al. 2019); however, cancers are prone to develop drug resistance, and there remains a lack of biomarkers for predicting this biological phenomenon (O’Sullivan et al. 2019; Dong et al. 2020). In non-small cell lung cancer (NSCLC), the KRAS-G12D mutation reduces secretion levels of the chemokines, CXCL10/CXCL11, in the tumor immune microenvironment (TIME) by targeting HMGA2 signaling, leading to a reduction in CD8^+^ tumor infiltrating lymphocytes, and thereby promoting the effects of anti-PD-1/PD-L1 immunotherapy (Liu et al. 2022). Paclitaxel-based chemotherapy can alleviate the TIME induced by KRAS-G12D mutation by upregulating HMGA2 signaling levels (Liu et al. 2022). Furthermore, the recent study demonstrated an interaction between lncRNA PiHL and enhancer of zeste 2 (EZH2) to promote HMGA2 expression, further mediated chemoresistance in colorectal cancer (Deng et al. 2021). Interestingly, concordant expression of HMGA2 and EZH2 proteins is important in lung metastasis of chemo-resistant TNBC (El Ayachi et al. 2019). Hence, HMGA2 has important roles in promoting BC drug resistance. When exposed to a 6 Gy radiation dose, Snail and HMGA2 expression levels were significantly upregulated in D7-6G cells, and TGF-βRI inhibitor treatment reversed the increase of D7-6 G cell proliferation (Yadav and Shankar 2019). The WNT inhibitor, ICG-001, reduces TNBC anthracycline sensitivity and restrains multi-organ metastases, such as those to liver, ovaries, and bone (Fatima et al. 2019). In summary, some small molecule inhibitors can be used to treat drug resistant BC by inhibiting HMGA2 expression.

## MiRNA influence on therapy response

MiRNAs strongly influence therapy response in BC. For example, miR-378a-3p and miR-378d expression in serum exosomes is increased in patients with BC receiving chemotherapy, while miR-378a-3p and miR-378d promote BC stemness and chemoresistance via activation of the EZH2/STAT3 signaling axis (Yang et al. 2021). Further, miRNA‐221 overexpression enhances BC resistance to adriamycin, thereby sustaining cell survival and invasion, by directly inhibiting PTEN expression and activating Akt/mTOR signaling (Yin et al. 2020). Endocrine therapy (tamoxifen), upregulates EREG in BC cells by downregulating miR-186-3p, and miR-186-3p inhibits EGFR signaling via targeting EREG in tamoxifen-resistant inT47D-TR cells, indicating that miR-186-3p is a promising therapeutic target in tamoxifen-resistant BC (He et al. 2019). Tamoxifen (TAM) resistance seriously affects therapeutic outcomes in BC. HMGA2 is clearly increased in tamoxifen resistant BC cell lines, while circTRIM28 restrained tamoxifen sensitivity by regulating miR-409-3p/HMGA2 signaling (Yang et al. 2022). Furthermore, the HMGA2 is highly expressed in 5-FU-resistant BC cells, while circFBXL5 promotes 5-FU resistance via the miR-216b/HMGA2 axis (Zhu et al. 2021). Similarly, down-regulation of the lncRNANEAT1, increased BC cell 5-FU sensitivity by regulating the miR-211/HMGA2 axis (Li et al. 2017). Current, new anti-cancer therapies based on miRNAs are under active development. The majority of research has shown that the miRNA/HMGA2 axis has important roles in tumor progression; however, there has been a lack of investigation into the role of miRNA/HMGA2 signaling in chemotherapeutic resistance of BC. Taken together, more in-depth exploration of miRNA therapy is needed, to reverse BC cell drug resistance by targeting HMGA2, and provide new BC treatment options.

## HMGA2 siRNA

Metastatic tumors, tumor recurrence, and drug resistance are challenges for standard treatment methods (e.g., surgery, chemotherapy, and radiation therapy) (Liyanage et al. 2019). For example, trastuzumab, a monoclonal antibody commonly used in the treatment of BC, is associated with toxic complications, including cardiac dysfunction, after long-term use (Zeglinski et al. 2011). Considering these adverse reactions to traditional treatment methods, regimen optimization is needed. Small interfering RNA (siRNA) is a key strategy for cancer treatment (Subhan and Torchilin 2023), where siRNAs target and silence RNAs by forming RNA-silencing complexes, leading to targeted RNA cleavage. Thus, siRNAs can specifically silence the expression of oncogenes, which is a major strategy in cancer therapy (El Moukhtari et al. 2023; Hu et al. 2020). HMGA2 siRNA can reduce cell viability and migration, as well as increase apoptosis of PC3 cells (Khajouee et al. 2022). Currently, the use of HMGA2siRNA has been proposed to improve proliferation and invasion ability of cancer (Liu et al. 2017); however, there remain many obstacles to the clinical translation of siRNAs, including low scalability, high cost, half-life, and short shelf life, which limit their large-scale use (Shahryari et al. 2021).

Nano-technology and drug delivery approaches have a major part to play in treatment of BC (Liyanage et al. 2019; Garbayo et al. 2020). Currently, numerous drugs delivery media are currently used for BC treatment, including liposomes, silica, viruses, and polymers, among others (Wang et al. 2021a, b, c; Behravan et al. 2022; Lohiya and Katti 2022). Methotrexate (MTX) is commonly used as auxiliary structure for modifying delivery systems (Álvarez-González et al. 2020), while poly-amidoamine dendrimers are frequently used polymeric nanoparticles. The current study demonstrated that the G4/MTX-siRNA nanocomplex targeting HMGA2 can significantly improve HMGA2 silencing efficacy, resulting in significant upregulation of apoptosis (Abedi Gaballu et al. 2021). Besides, in gastrointestinal cancer cells, HMGA2 siRNA-loaded nanoliposomes increased the expression of caspase3 and caspase 9, and decreased that of BCL2, leading to reduced apoptosis (Mohammadi et al. 2019). From this, it can be seen that nanomaterials as carriers for delivering HMGA2 siRNA are a potential strategy for treating tumors. Various drug delivery systems are currently used to treat tumors, including polymeric nanoparticles (Zou et al. 2019), metallic nanoparticles (Cho et al. 2019), magnetic nanoparticles (Pan et al. 2007), carbon-based nanoparticles (Ringel et al. 2014), liposomes and lipid nanoparticles (Medler et al. 2019), and dendritic polymers (Zou et al. 2019; Ambrosio et al. 2020; Jain et al. 2021; Mirza and Karim 2021). Mounting evidence suggests that nanoparticles can achieve better penetration and improve drug bioavailability, through both passive and active targeting methods (Jain et al. 2021). Therefore, more research is needed to optimize drug delivery systems and stimulate HMGA2 siRNA release. Furthermore, various forms of inorganic nanoparticles stimulate drug release in BC, including systems using magnetism-, laser-, and ultrasound-based methods, among others (Mack et al. 2004; Feril et al. 2021; Mokoena et al. 2021). Therefore, use of inorganic nanoparticles to target and promote HMGA2 siRNA release is a potential method to treat BC. Exploring a strategy to combine organic and inorganic nanoparticles is another new direction with potential to achieve more efficient stimulation of HMGA2 siRNA release.

## Future perspectives and conclusion

With respect to therapeutic targets, deeper understanding of molecular mechanisms involved in BC has led to the identification of specific targets (Velikyan 2020), which can be used to diagnose and treat individuals, thereby improving treatment efficacy (Jokar et al. 2021). Evidence from the literature and clinical studies demonstrates that HMGA2 is expressed at high levels in patients with BC and BC cell lines. Further, HMGA2 overexpression is clearly positively correlated with poor prognosis in patients with BC. The Wnt signaling pathway-related protein, β-catenin, prevents HMGA2 transcription, inhibiting its activation and expression levels, thereby suppressing BC cell growth, invasion, and metastasis; however, it is unclear whether HMGA2 is involved in other signaling pathways that promote BC development. Therefore, it is crucial to study molecules that target or interact with HMGA2, to gain an in-depth understanding of the pathological characteristics of BC.

In addition to protein molecules, multiple non-coding RNAs (lncRNAs, circRNAs, and miRNAs) regulate HMGA2 expression to promote BC cell EMT, metastasis, and drug resistance. Furthermore, miRNA can indirectly inhibit HMGA2 expression by regulating its transcription activity (Mansoori et al. 2021). In most cases, lncRNAs control HMGA2 expression through sponging miRNAs, while circRNAs can also promote HMGA2 signaling but, similar to lncRNAs, mainly increase HMGA2 levels by targeting miRNAs. Notably, HMGA2 expression in BC is regulated by circRNAs and lncRNAs, which are both tumor promoting factors, and their mechanisms of action involve enhancing HMGA2 expression through sponging miRNAs. Future experiments should focus on clarifying the regulatory effect of tumor suppressor lncRNAs and circRNAs on HMGA2 in the context of BC. Furthermore, research to date has shown that HMGA2 siRNA can effectively inhibit HMGA2 expression, thus promoting tumor cell apoptosis, and inhibiting BC cell growth, proliferation, migration, and drug resistance. Nevertheless, improvements in treatment efficiency are required; for example, by combining biocompatible and safe nanomaterials as carriers, to efficiently deliver HMGA2 siRNA to treat BC. Given the key role of HMGA2 in regulating the progression of BC and other tumors, and influencing drug resistance, further investigations are warranted to develop effective HMGA2 inhibitors, which will be of significance for BC treatment in the clinic.

## Data Availability

No data was used for the research described in the article.

## Abbreviations

HMGA2: High mobility group AT-hook 2
HMG: High mobility group
BC: Breast cancer
ER: Estrogen receptor
PR: Progesterone receptor
HER2: Human epidermal receptor 2
TNBC: Triple-negative breast cancer
CSC: Cancer stem cell
TIME: Tumor immune microenvironment
LncRNA: Long non-coding RNA
CircRNA: Circular RNA
miRNA: MicroRNA
EMT: Epithelial-mesenchymal transition
TME: Tumor microenvironment
siRNA: Small interfering RNA
MTX: Methotrexate
m6A: N6-methyladenine
NSCLC: Non-small cell lung cancer
